# Legal Basis and Regulatory Requirements for Utilizing Real‐World Evidence in Medical Product Regulatory Decision‐Making

**DOI:** 10.1002/pds.70373

**Published:** 2026-04-16

**Authors:** Sungmin Park, Euna Han

**Affiliations:** ^1^ Graduate School of Public Health, Seoul National University Seoul South Korea; ^2^ College of Pharmacy, Yonsei Institute of Pharmaceutical Sciences, Yonsei University Incheon South Korea

**Keywords:** legal basis, medical product, real‐world evidence, registry, regulatory decision‐making

## Abstract

- Real‐World Evidence (RWE) application in regulatory decisions remains limited.
- Clear standards are needed for data quality, transparency, and acceptable study designs for the use of RWE in regulatory contexts.
- Registry certification system and research plan review system for RWE research would help reduce regulatory uncertainty.
- To balance data protection and research utility, relaxation of privacy protection regulations for designated government‐managed registries could be considered.

## The Plain Language Summary

There is a lack of a clear system for using real‐world evidence as an alternative to randomized controlled trials in regulatory decisions. We propose a registry certification and research plan review system, accompanied by conditional relaxation of privacy regulations, to ensure the reliability of real‐world evidence and support its effective regulatory application.

## 1 Introduction

Real‐world data (RWD) refers to data collected from everyday settings rather than from controlled randomized controlled trials (RCTs). Examples of RWD are health records, claims and billing data, pharmacy dispensing data, disease or product registries, laboratory and diagnostic test results, and data generated from routine clinical practice. Real‐world evidence (RWE) refers to clinical evidence generated through the processing and analysis of RWD [1]. Internationally, the use of RWE is increasing across various aspects of regulatory decision‐making throughout the entire lifecycle of medical products, from pre‐market approval to post‐market surveillance [2]. However, using RWE as a partial or full alternative to RCTs in regulatory decision‐making is uncommon and is typically limited to circumstances in which RCTs are impractical or unethical (e.g., rare diseases). The key challenge is not the technical feasibility of using RWE per se, but the regulatory conditions under which RWE is considered acceptable evidence for a given decision context. Although research using RWD is encouraged, RCTs remain the primary evidentiary standard for evaluating medical product safety and efficacy, even in US or EU [3, 4]. This study explores prerequisites to establish a medical product safety management system that supports the effective use of RWE instead of RCTs, focusing on the existing regulatory system in Korea. This study provides insights not only for Korea but also for countries considering the use of RWE as a partial or full alternative to RCTs. Among the various types of RWD, this paper focuses on registries, which could offer a level of reliability comparable to that of RCTs.

## 2 How the Existing Regulatory System in Korea Ensures the Reliability of Evidence

### 2.1 Existing Regulatory System for Ensuring the Reliability of Evidence for RCTs or Non‐Clinical Trials

Currently, standards for data quality, transparency, and reliability for the use of RWE in place of RCTs in regulatory contexts are unclear. Consequently, companies avoid conducting RWE studies for regulatory decisions, which limits opportunities for regulatory authorities to review them. Therefore, we reviewed current measures to ensure data quality, transparency, and reliability in RCTs and non‐clinical trials (*Pharmaceutical Affairs Act*, Article 2, “non‐clinical study” means a study conducted through the use of animals, plants, microorganisms, physical or chemical mediums, or the components thereof in the same conditions as those in a laboratory, so as to obtain various data on the nature or safety of study materials which influence the health of humans) in regulatory decision‐making. We explored ways to apply these measures to RWE to ensure that RWE is sufficiently reliable to be used in regulatory decisions.

### 2.2 Good Clinical Practice for RCTs

In Korea's regulatory system, RCTs are used for regulatory decision‐making to obtain reliable results and to protect trial participants' rights and confidentiality [5]. Good clinical practice (GCP) is an international ethical and scientific quality standard of RCTs. Regulatory authorities use GCP to verify the reliability of data generated during RCTs and to protect trial participants. GCP includes regulations to ensure that trial participants' privacy is protected [6].

### 2.3 Clinical Trial Institution Designation System for RCTs

Another regulatory measure to ensure the quality and reliability of RCTs in Korea is the Clinical Trial Institution Designation System (*Pharmaceutical Affairs Act*, Article 34‐2). To be designated as a Clinical Trial Institution, a healthcare institution must meet specific standards regarding its facilities, specialized personnel, and equipment. The Korean regulatory authority, the Ministry of Food and Drug Safety (MFDS), evaluates designation applications through an on‐site inspection using an official rubric (Safety Regulations for Pharmaceuticals, Article 34‐4).

### 2.4 Clinical Trial Protocol Approval System for RCTs

Additionally, the Clinical Trial Protocol Approval System (*Pharmaceutical Affairs Act*, Article 34) requires those seeking approval for RCTs to submit a development plan, pharmaceutical manufacturing and quality control standards, non‐clinical trial data, clinical trial institution information, informed consent forms, and the clinical trial protocol. The protocol must include a summary, objectives, study design, participant criteria, evaluation procedures, data analysis methods, ethical considerations, and safety measures (*Pharmaceutical Safety Regulations*, Article 24).

### 2.5 Non‐Clinical Trial Institution Designation System for Non‐Clinical Trials

The Non‐Clinical Trial Institution Designation System in Korea (*Pharmaceutical Affairs Act*, Article 34‐3) was introduced to ensure the reliability of non‐clinical trial results (*MFDS's Good Laboratory Practice Notification*). To be designated as a Non‐Clinical Trial Institution, a laboratory institution must meet requirements for facilities, personnel, and equipment specific to each type of non‐clinical trial. The institution must also establish systems for trial management and reliability assurance and undergo regular bi‐annual inspections by MFDS (*Non‐clinical Trial Management Standards*, Articles 4, 39). However, non‐clinical trials can be performed at research labs that are not designated as non‐clinical trial institutions, and their results can still be used in regulatory decision‐making. Researchers conducting non‐clinical trials are not required to submit a trial protocol in advance for approval by MFDS. This distinction exists because non‐clinical trials, unlike RCTs, involve no trial participants. Therefore, no legally mandated standards regarding research design and participant protection are needed.

## 3 Prerequisites for RWE Use in Regulatory Decision‐Making for Medical Products in Korea

### 3.1 The Need for Practical Standards for RWE Research That Are Comparable to GCP

If RWE studies are used to replace or complement RCTs, their data quality, transparency, and reliability must be upheld in line with the standards used for RCTs [7]. Therefore, policymakers must set standards for data quality management and outline how RWE research reliability and transparency should be ensured. Guidelines should clarify which data are needed and how the available RWD can be assessed for their potential to meet the study objectives [8]. For registries or structured data‐collection systems, guidelines should specify which data elements to collect and how to collect them.

Although the FDA [9] and EMA have both established relevant guidelines [8], it remains difficult for researchers to determine—based on current FDA or EMA guidelines—whether the RWD they intend to use or the RWE study they plan to conduct has the level of quality or reliability necessary to serve as an alternative to RCTs. Moreover, no such guidelines are yet available in Korea. Consequently, companies cannot determine which RWD to use, nor how to conduct RWE studies to support the regulatory decision‐making of MFDS [10]. As a result, few researchers are willing to initiate RWE studies, even though reliable RWE, once generated, could be utilized in regulatory decisions. Other countries appear to face similar situations [3, 4].

### 3.2 Registry Certification System for RWE Research Comparable to Those for RCTs or Non‐Clinical Trials

Registries have relatively high reliability among various types of RWD for medical product regulatory decision‐making [11]. Therefore, we suggest starting with registries to establish a system to generate RWE that can replace or complement RCTs. A key design choice is whether certification should apply to the institution operating registries or to each registry itself. Because a single institution may operate multiple registries using different data elements, collection workflows, and quality controls, a regulator may reasonably prefer a registry‐level assessment. We therefore propose a two‐tier approach: (i) certification of the registry operating organization's cross‐cutting capabilities (e.g., governance, quality management system, privacy, and security controls), and (ii) certification of individual registries based on their specific data elements, collection processes, and quality performance.

To make certification meaningful and reviewable, registry certification could focus on a limited number of domains, such as [7, 10]: (1) governance and transparency (purpose, oversight, and data access policies); (2) standardized data elements and collection workflows (data dictionary, SOPs, and staff training); (3) quality management and performance monitoring (completeness, consistency, timeliness, query resolution, and audits); (4) data provenance, traceability, and change control (audit trails, versioning, and system validation); (5) patient protections and ethical oversight (Institutional Review Board (IRB) or Independent Ethics Committee (IEC) processes, and consent or opt‐out mechanisms); and (6) privacy and security controls (pseudonymization, encryption, role‐based access, access logs, and incident response) (Table 1).

**TABLE 1:** Comparison between the current Korean systems for clinical and non‐clinical trials and our proposed system for RWE research.

Regulatory element	RCTs	Non‐clinical trials	Proposed registry‐based RWE
Institution qualification	Required	Optional	Optional two‐tier^1^
Who can conduct studies?	Restricted	Not restricted	Not restricted
Prior protocal or plan submission	Required	Not required	Optional plan review^2^
Primary purpose	Participant protection and reliability	Lab reliability assurance	Reduce uncertainty via standards and privacy protection

^1^ Two‐tier: registry operating institution (governance, quality management system, privacy and security controls) and registry (data elements, collection processes, quality performance).

^2^ Plan review checks minimum requirements and is non‐binding for regulatory endorsement (optional scientific advice may be offered).

### 3.3 Research Plan Review System Comparable to Clinical Trial Protocol Approval System for RCTs

Additionally, a registry research protocol review mechanism could be introduced, analogous to clinical trial protocol submission, allowing registry researchers to submit prespecified study plans for a structured review. This review would primarily assess whether a protocol meets minimum requirements for transparency, data provenance, patient protections, and privacy or security safeguards. It would not constitute a binding regulatory endorsement that a specific comparator, exposure window, or statistical model is optimal. To further reduce uncertainty, an optional non‐binding scientific advice pathway could provide early feedback on key design choices (e.g., comparator selection, confounding control strategy, outcome definitions, and follow‐up period).

## 4 Proposed Certification System and Research Plan Review System

Korean law prohibits undesignated clinical trial institutions from conducting RCTs and also prohibits any RCTs from being conducted without prior approval. In contrast, it does not restrict undesignated non‐clinical trial institutions from performing non‐clinical trials, nor do these trials require prior approval. Likewise, registry research by non‐certified registry operating institutions or non‐certified registries, and studies conducted without plan review should still be accepted for regulatory decision‐making if data quality and reliability are adequately demonstrated. The systems for registry certification or research plan review should function as supportive mechanisms that define quality standards and reduce the transaction costs of identifying trustworthy registries. Providing a clear certification and plan review pathway can reduce regulatory uncertainty and promote confidence in registry data. These supportive pathways would not replace case‐by‐case “fit‐for‐purpose” assessment for the specific regulatory question.

### 4.1 Regulatory Exemptions for Privacy Restrictions in Government‐Designated Registries

A practical barrier to generating large‐scale, long‐term RWE is the requirement to obtain individual informed consent whenever identifiable data are used. In many real‐world settings, re‐consenting large cohorts is logistically infeasible and may introduce selection bias, undermining both feasibility and validity. Therefore, policy proposals should explicitly address consent and lawful basis under strict governance.

The protection of research subjects' rights in RWE research must be ensured through robust measures to protect privacy [12]. Under Korean privacy law, RWD study involving personally identifiable information is not permitted without obtaining informed consent (*Korean Personal Information Protection Act*, Article 15, 17). RWD studies that do not involve the collection of personally identifiable information may be conducted without informed consent (*Korean Personal Information Protection Act*, Article 58‐2, 28‐2). However, in many cases, generating regulatory‐grade evidence requires RWD that include personally identifiable information. We suggest that regulatory burdens can be reduced by conditionally establishing targeted legal pathways for consent and privacy for registries that serve public interest or meet high‐level data protection standards. This may include designating qualified registries and granting limited exemptions from privacy regulations while maintaining strong governance and operational oversight.

For example, long‐term follow‐up studies for implantable medical devices, advanced biopharmaceuticals, or conditional approvals may be eligible for such flexibility when managed under strict data management systems required by regulatory authorities. Since 2024, Korea has operated “Data Innovation Zones” to allow safer and more flexible use of pseudonymized data, which may offer a useful model. The zones manage the entire data‐processing workflow and enhance security through dedicated expert units and real‐time recording systems (*Press Release, Personal Information Protection Commission, 2025. 9. 17*.). Privacy regulations that require researchers to obtain individual patient consent (*Korean Personal Information Protection Act*, Article 15, 17) and human subject research regulations that require obtaining IRB approval (*Bioethics and Safety Act*, Article 15) may be exempted. These registries, however, should remain subject to regular audits to verify compliance with infrastructure and data protection requirements. To safeguard patient rights, an opt‐out mechanism similar to that of NHS Digital may be considered. While data minimization or pseudonymization are important safeguards, some designated registries may still require controlled use of identifiable information for linkage and long‐term follow‐up; in such cases, stricter governance, access controls, and auditability become even more critical.

## 5 Conclusion

In summary, under the current medical product regulatory system, the use of RWE in regulatory decision‐making is possible but rare. We reviewed the existing regulatory system in Korea, which ensures the reliability of clinical trial and non‐clinical trial evidence. A similar system could be applied to RWE research. Registry certification and research plan review systems, both of which researchers may voluntarily and optionally choose to use, may help mitigate regulatory uncertainty. Special provisions to enable feasible consent or privacy pathways under strict governance may be considered in cases with high public interest or specially managed privacy protection systems. A bill to promote the use of health and medical data was introduced in the National Assembly of Korea in November 2024. The bill aims to ensure the safe utilization of health data and to foster research by designating “medical data‐centered hospitals,” whose collected data may be used for government‐led health data initiatives. If enacted, the policy proposals discussed in this paper could be implemented at these designated hospitals. Implementing this system would necessitate additional legislative amendments. Further research and discussion on this topic (e.g., establishing standards for certification or plan review) are expected in the future.

## Author Contributions

S.M. Park and E. Han wrote the manuscript. E. Han designed the research.

## Funding

This work was supported by the Korea Ministry of Food and Drug Safety and Seoul National University.

## Conflicts of Interest

The authors declare no conflicts of interest.
